# Coronary-to-pulmonary artery fistula in a patient with atypical chest pain: case presentation and literature review

**DOI:** 10.1007/s00276-025-03724-7

**Published:** 2025-10-21

**Authors:** Andrei Constantin Rusali, Caterina Ioana Lupu, Mihaela Macrina Manolache, Lavinia Maria Rusali, Petru Bordei, Lucia Cojocaru

**Affiliations:** 1 Department of Cardiology, Constanta County Clinical and Emergency Hospital, 145 Tomis Boulevard, 900591 Constanta, Constanta County Romania; 2 Faculty of Medicine, Ovidius University of Constanta, University Avenue No. 1, 900470 Constanta, Constanta County Romania

**Keywords:** Coronary-to-pulmonary artery fistula, Coronary artery fistula, Congenital heart defect, Computed tomography coronary angiography, Conservative management, Case report

## Abstract

Coronary artery fistulas are rare anomalies characterized by abnormal communications between a coronary artery and a cardiac chamber, vein, or great vessel. This review explores the pathophysiology, clinical presentation, diagnostic modalities, and management of CPAFs, with a focus on their hemodynamic implications. A clinical case is presented to illustrate the diagnostic process and decision-making in the management of CPAFs. The patient was admitted into hospital for chest pain and an abnormal flow in the pulmonary artery observed on transthoracic echocardiography in the emergency department. Further investigations revealed the patient had a coronary-to-pulmonary fistula connecting the left anterior descending artery to the pulmonary artery trunk. Myocardial perfusion scan was performed and it demonstrated normal perfusion in the left anterior descending artery territory. Different causes for chest pain were investigated—upper digestive endoscopy revealed peptic ulcer, which was successfully treated. This review underscores the importance of individualized approaches to treatment and highlights the potential for conservative management in selected patients.

## Introduction

Coronary artery fistulas are abnormal communications between a coronary artery and a cardiac chamber, vein, or great vessel. Coronary artery fistulas are a rare finding in general population, with a prevalence of 0.9% [1]. Based on standard coronarography, the original prevalence was around 0.05–0.25%, but after the introduction of computerized tomography coronarography the prevalence raised to 0.9%. From an anatomical standpoint, the right coronary artery is the most frequent origin for a coronary artery fistula (50–55% of cases), followed by the left anterior descending artery (LAD) (35–40% of cases) and, lastly, the left circumflex artery (5–20% of cases) [2].

Coronary-to-pulmonary artery fistulas (CPAF) are a subset of these anomalies, which involve an aberrant connection between a coronary artery and one of the pulmonary arteries. First described by Krause in 1865 [2], CPAF remains an uncommon condition, with an estimated prevalence of 0.1–0.2% among individuals undergoing coronary angiography. Among coronary artery fistulas, CPAFs account for 15–30% of cases [2].

## Methods

We used medical databases (PubMed, Cochrane Library and Europe PMC), using a combination of keywords, synonyms and Boolean operators: ("Coronary to pulmonary artery fistula" OR "Coronary artery fistula" OR "Coronary arteriovenous fistula") AND (Etiology OR Diagnosis OR Management OR Treatment OR Imaging OR Intervention).

The selected clinical case highlights the process of differential diagnosis of chest pain in a patient with a coronary-to-pulmonary fistula which was discovered during routine echocardiography.

## Literature review

### Epidemiology

Coronary fistulas are a rare anomaly of the coronary arteries. Data from the international literature indicate a prevalence of ~ 0.05 to 0.25% on conventional coronary angiography, but the reported frequency has increased to ~ 0.75 to 0.9% in studies using CT angiography, suggesting that non-invasive techniques can also identify small, previously undetected fistulas [1]. Overall, it is estimated that only about 1 in 50,000 people in the general population has a documented coronary fistula [3]. Coronary fistulas constitute almost half (≈ 50%) of all congenital anomalies of the coronary arteries [4]—underlining their importance in the context of congenital cardiology. A study conducted in a high-volume cardiovascular center in Romania (C.C. Iliescu Institute, 2014) reported coronary anomalies in 0.53% of the ~ 5800 patients investigated coronary angiography in one year, with coronary arteriovenous fistulas representing ~ 13% of these anomalies [5]

Demographically, coronary fistulas occur in both men and women, with no gender or race predilection [6, 7]. They can be diagnosed at any age: from newborns (in the context of complex cardiac malformations) to the elderly. However, the presentation differs depending on age. In childhood, large fistulas may cause symptoms of heart failure or may be detected due to a continuous heart murmur heard on clinical examination. However, most children with small fistulas remain asymptomatic. Studies show that < 20% of patients under 20 years of age with coronary fistulas present symptoms, while over 60% of patients over 60 years of age become symptomatic [8].

Regarding the anatomical epidemiological features, in ~ 80 to 90% of cases the fistula is solitary (involving a single coronary artery of origin and a single fistulous tract) [4]. Multiple fistulas (involving both coronary arteries or multiple drainage tracts simultaneously) are rarer, occurring in ~ 10 to 20% of cases [9].

### Etiology

Approximately 80–90% of coronary fistulas are congenital [7], resulting from abnormal embryological development of the coronary circulation. Acquired coronary fistulas are much rarer (≈10% of all) and occur secondary to external factors that create an abnormal communication between a coronary artery and neighboring structures. Penetrating chest trauma is one cause—for example, gunshot or stab wounds that damage the wall of a coronary artery and a cardiac cavity, forming a fistulous connection [10]. Similarly, severe nonpenetrating chest trauma (sudden decelerations) has occasionally been implicated.

In the current medical era, a significant proportion of acquired fistulas are of iatrogenic origin. Various cardiovascular interventions can generate coronary fistulas as an undesirable side effect: cardiac surgery (e.g. coronary-chamber fistulas after aorto-coronary bypass, after correction of tetralogy of Fallot or after valvular procedures) and percutaneous interventional procedures (PTCA coronary angioplasty, radiofrequency ablation for arrhythmias, endomyocardial biopsies—especially in transplant patients, implantation of pacemakers. For example, post-PTCA coronary-chamber fistulas are described by coronary artery dissection with communication to the cavity, or coronary-ventricular fistulas after surgical repair of cardiac defects. The exact incidence of iatrogenic fistulas is difficult to determine, but the increase in the number of cardiovascular procedures has made such cases more common in recent decades [11].

More rarely, coronary fistulas may arise as a result of intrinsic pathological processes: for example, fistulas have been reported to develop during the healing phase after myocardial infarction (communication between an infarcted coronary artery and the ventricular cavity) or in the context of vasculitis (inflammatory lesions that erode the vascular wall). These mechanisms involve neovascularization and “recanalization” with the formation of shunt vessels. Although uncommon, such situations should be considered especially in patients with compatible antecedents (extensive infarction, Kawasaki disease, etc.) [11].

### Pathophysiology

From a pathophysiological point of view, the coronary fistula creates an abnormal shunt that allows high-pressure blood from the coronary artery to be diverted to a lower pressure area (cardiac chamber or venous vessel) bypassing the myocardial microcirculation [12].

The main consequence is the reduction of myocardial perfusion in the territory irrigated distal to the fistula, a phenomenon known as “coronary steal”. The severity of this steal depends on the caliber of the fistula and the resistance of the drainage segment: a large shunt will constantly “steal” a significant portion of the coronary nutrient flow, while a small shunt may not produce ischemia except under stress conditions. Two forms of coronary steal are described:

- *Persistent (resting) steal*: occurs in large fistulas with very low resistance at the drainage end, where a significant volume of blood is continuously diverted through the fistula. This “short-circuited” flow deprives the myocardium of the appropriate portion of the nutrient blood flow, which can cause myocardial ischemia even at rest. Chronic ischemia can lead to segmental myocardial dysfunction (including papillary muscle dysfunction—causing valvular regurgitation) and the development of collaterals. Large fistulas also tend to cause dilation of the proximal coronary artery (ectasia/aneurysm) due to increased flow. An extreme case reported is the spontaneous rupture of such an aneurysmal fistula with acute cardiac tamponade, although cases are exceptionally rare [13]
- *Episodic (effort) steal*: occurs in fistulas of moderate size, which under basal conditions do not cause significant ischemia, but during physical exertion or other conditions with increased myocardial oxygen demand, preferential flow to the fistula limits the corresponding increase in perfusion through the microcirculation, leading to angina pectoris and transient ischemic changes. Basically, during exertion, normal coronary vessels dilate compensatory, but the fistula, lacking capillary resistance, takes over a disproportionate portion of the flow, leaving the myocardium under perfused. This type of steal is difficult to objectify with conventional ischemia tests, because pharmacological vasodilators (adenosine) also dilate the fistula, accentuating the steal and making it difficult to assess the degree of ischemia. However, the presence of exertional angina in a patient with a coronary fistula is an important indication of hemodynamic significance.

In addition to ischemia, another major hemodynamic effect is given by the volume of the shunt and the consequences on cardiac load. In essence, most coronary fistulas are left-to-right shunts (coronary arterial blood reaches the venous circulation/right cavities). If the volume of the shunt is large and long-lasting, it can result in volume overload of the right heart, its dilation and over time pulmonary hypertension and high-output heart failure. Fistulas draining into the pulmonary artery, right atrium or right ventricle behave physiologically similar to other left-to-right shunt defects (e.g. PCA, septal defect)—if the shunt volume exceeds 30–50% of the systemic flow, symptoms of heart failure, cardiomegaly and signs of PH may occur. In contrast, left-to-left fistulas (e.g., a fistula from the ADA into the left ventricle) do not produce volume shunting between different circulations, but they can still cause local overload of the left ventricle and contribute to high cardiac output, placing additional strain on the heart. In both situations of significant hemodynamic shunting, the heart can eventually develop high-output heart failure with dilated chambers, tachycardia, and shunt cardiomyopathy. It is important to note that coronary fistulas tend to enlarge progressively over time. Even if a patient is asymptomatic at 20–30 years of age, their fistula may increase in size decades later, thus increasing the shunt volume and risk of complications [8, 14].

Co-existing atherosclerotic coronary artery disease may further complicate the pathophysiology. If the fistula arises proximal to a coronary stenosis, distal myocardial ischemia is predominantly driven by the phenomenon of steal (left-to-right shunt) and post-stenotic perfusion pressure decrease [15]. Conversely, if the fistula is distal to a significant coronary lesion, reverse shunting (right-to-left at the microvascular level) may occur—meaning that relatively deoxygenated blood from the drainage chamber (e.g., the right ventricle) may flow retrogradely through the fistula across the lesion, mixing with oxygenated coronary blood and exacerbating ischemia distal to the stenosis.

Potential complications of untreated coronary fistulas derive from the above mechanisms: chronic myocardial ischemia can lead to angina pectoris or infarction, volume overload can lead to cardiac dilation, chronic local hypoxemia can cause myocardial fibrosis and arrhythmias. In addition, the fistula area (having turbulent flow) predisposes to the development of intravascular thrombi and infective endarteritis/endocarditis along the fistula tract. Bacterial endocarditis is a described complication, which is why antibiotic prophylaxis is recommended in patients with coronary fistulas in high-risk situations (dental procedures, etc.) [16].

### Clinical presentation

Most CPAFs are asymptomatic and are discovered incidentally during routine medical check-ups. The likelihood of an incidental diagnosis has risen dramatically with the advent and widespread adoption of advanced imaging technologies. While conventional invasive coronary angiography (CAG) historically identified coronary artery fistulas (CAFs) in 0.05% to 0.25% of patients, the superior spatial resolution of computed tomography coronary angiography (CTCA) has unveiled a much higher prevalence, estimated to be as high as 0.9% [17]. This suggests the existence of a large, previously under-recognized population with asymptomatic fistulas.

In these patients, the fistula is typically small, and the left-to-right shunt is hemodynamically insignificant, meaning the cardiovascular system can readily accommodate the minor volume load without producing overt symptoms [18]. However, the absence of symptoms does not equate to an absence of risk. The underlying pathophysiological mechanisms of coronary steal and potential for chamber overload remain, albeit at a subclinical level [18]. The primary clinical challenge in this cohort is therefore one of risk stratification: identifying which asymptomatic fistulas are likely to progress and cause future complications, thereby warranting closer surveillance or consideration for pre-emptive closure.

When CPAFs become hemodynamically significant, they produce a constellation of symptoms that directly reflect the underlying pathophysiology. In a large review of adult patients with CAFs, 85% were found to be symptomatic [19]. Symptomatic cases may present with angina pectoris, heart failure, arrhythmias, hemoptysis and even endocarditis: a rare but serious complication [20–23]. The onset and severity of these symptoms are governed by the magnitude of the shunt, which can lead to myocardial ischemia, volume overload of the right heart, arrhythmias, and other serious complications [17].

Symptoms are more common in older patients or those with large fistulas. Sometimes, a murmur can be heard in the second intercostal space, left of the sternum, with a systole-diastolic crescendo-decrescendo pattern [2].

Dyspnea, particularly on exertion, is the most common presenting complaint in patients with symptomatic CPAF, reported in 31% to nearly 49% of cases [19]. It is often accompanied by generalized fatigue and reduced effort tolerance [17]. These symptoms are the direct clinical expression of a significant left-to-right shunt. The diversion of blood from the high-pressure coronary artery into the low-pressure pulmonary artery increases pulmonary blood flow, imposing a chronic state of volume overload on the right-sided cardiac chambers [24]. Over time, this leads to dilation and dysfunction of the right ventricle, culminating in right-sided heart failure. The patient’s exertional capacity becomes progressively limited as the compromised right ventricle is unable to adequately augment its output to meet the body’s increased metabolic demands during physical activity [25].

Angina pectoris or non-specific chest pain is the second most prevalent symptom, affecting 21% to 23% of patients [19]. The character of the pain can be typical of exertional angina or may be atypical, presenting a diagnostic challenge [26]. The primary mechanism responsible for this symptom is the “coronary steal phenomenon” [27]. The fistula constitutes a low-resistance pathway that diverts, or “steals,” a significant portion of the coronary blood flow away from the high-resistance myocardial capillary network that lies distal to the fistula’s origin [24]. This shunting of blood results in a state of functional myocardial ischemia, producing angina even in the complete absence of obstructive atherosclerotic coronary artery disease (CAD). Consequently, CPAF must be considered in the differential diagnosis for any patient, particularly younger individuals, who presents with rrhyt symptoms but is found to have non-obstructed coronary arteries on angiography [28].

Palpitations are another common complaint, reported in approximately 13% of symptomatic patients [23]. These symptoms arise from the electrophysiological consequences of the fistula’s hemodynamic burden. The chronic volume and pressure overload imposed on the cardiac chambers—primarily the right atrium and ventricle—leads to progressive chamber dilation [29]. This structural remodeling creates an arrhythmogenic substrate. Atrial fibrillation is a well-documented complication, occurring in about 5% of patients [19]. Furthermore, ventricular arrhythmias, ranging from premature ventricular contractions (PVCs) and non-sustained ventricular tachycardia (NSVT) to life-threatening sustained ventricular tachycardia, have been reported and can be a presenting feature [17].

If left uncorrected, large CPAFs can lead to a number of severe, late-stage complications:

- Congestive Heart Failure (CHF): This is the ultimate hemodynamic consequence of a large, persistent left-to-right shunt and is reported in approximately 8% of adult patients [17].
- Infective Endocarditis (IE): A rare (4% of cases) but devastating complication. The high-velocity, turbulent jet of blood entering the pulmonary artery or a cardiac chamber can damage the endothelial surface, creating a non-bacterial thrombotic vegetation that serves as a nidus for infection. This risk is particularly associated with coronary-cameral fistulas (draining into a heart chamber) [17, 19, 30].
- Myocardial Infarction (MI): While uncommon, MI can occur as a result of profound coronary steal or, more rarely, from thrombosis and embolization originating within the fistula or an associated aneurysm. At least one case has been reported to present as a non-ST elevation myocardial infarction (NSTEMI) secondary to the steal phenomenon [17, 18].
- Aneurysm Formation and Rupture: The incessant high-flow state and turbulence within the feeding coronary artery can lead to progressive aneurysmal dilatation, a finding noted in about 14% of cases. While spontaneous rupture is a rare event, it is often catastrophic, leading to hemopericardium and cardiac tamponade [17, 30].

### Diagnosis

The physical examination can provide crucial clues to the presence of a CPAF. The most suggestive and common physical finding is a continuous cardiac murmur, which is audible in up to 82% of symptomatic patients [17].

The murmur is audible throughout both systole and diastole, and may exhibit a crescendo-decrescendo pattern [19, 26]. This continuous nature is a direct result of the persistent pressure gradient between the high-pressure coronary artery and the low-pressure pulmonary artery that exists throughout the entire cardiac cycle.

The location of the murmur on the precordium can serve as a valuable diagnostic clue to the fistula’s termination site [31]. For a CPAF draining into the main pulmonary artery, the murmur is classically best appreciated at the upper left sternal border, typically in the second or third intercostal space. In contrast, fistulas draining into the right atrium may produce a murmur heard best to the right of the sternum, while those terminating in the right ventricle are often loudest at the lower left sternal border [32]. In patients with large shunts and advanced disease, the examination may also reveal signs of right heart failure, such as elevated jugular venous pressure, peripheral edema, or hepatomegaly [17].

The diagnosis of coronary-to-pulmonary fistulas relies on multiple imaging modalities that delineate the fistula’s anatomy, hemodynamics and impact: transthoracic echocardiography (TTE), transesophageal echocardiography (TEE), coronary angiography, computed tomography angiography (CTA), magnetic resonance imaging (MRI) and myocardial perfusion scan.

The chest radiograph is frequently unremarkable in patients with CPAF [32]. In cases with a large shunt, however, it may show non-specific signs of cardiac involvement. These include cardiomegaly due to chamber enlargement and increased pulmonary vascular markings (shunt vascularity) reflecting the augmented pulmonary blood flow. In a review, an abnormal shadow on the chest X-ray, often related to a large aneurysmal fistula, was noted in only 4% of patients [19].

CPAF are hard to visualize on catheter coronary angiography (CAG). Moreover, coronarography is limited due to its 2-dimensional projection. It cannot demonstrate the blood vessel network surrounding the fistula and it cannot show other anatomical relationships between the fistulous vessel and the other structures of the heart. While coronarography’s role as the primary diagnostic tool has been largely superseded by computer tomography coronary angiography (CTCA), it remains indispensable in several scenarios. Its current role is to:

- Confirm the diagnosis when non-invasive tests are conflicting or non-diagnostic.
- Simultaneously evaluate for the presence of concomitant atherosclerotic CAD, which is a common consideration in older patients presenting with chest pain [19].
- Serve as the imaging platform to guide percutaneous transcatheter closure, providing real-time visualization of catheters, wires, and closure devices [33].

In cases where the ischemic consequence of a CPAF is uncertain, intracoronary functional assessment can be invaluable. Fractional Flow Reserve (FFR) is an emerging invasive technique employed during coronarography [30]. This procedure involves advancing a specialized pressure wire into the coronary artery, positioned distally to the origin of the fistula. FFR directly quantifies ischemia caused by the steal phenomenon by measuring the pressure drop across the myocardial territory.

Transthoracic echocardiography (TTE) has the advantage of generating 3D images but, it is limited in visualizing the coronary arteries and the fistulous vessel. TTE can visualize the abnormal flow entering the pulmonary artery using color flow Doppler imaging, and it can provide essential information about possible comorbidities. A saline contrast study may also be performed, with a positive result indicating the presence of a left-to-right shunt [18]. Beyond identifying the shunt, TTE plays a critical role in assessing its hemodynamic consequences. It allows for the evaluation of right atrial and ventricular dilation, a direct measure of the chronic volume overload. It can also be used to assess right ventricular systolic function and, by measuring the peak velocity of the tricuspid regurgitation jet, to estimate the systolic pulmonary artery pressure [24]. The main limitation of TTE is its frequent inability to visualize the entire anatomical course of the coronary arteries and the fistulous tract itself, particularly the origin and distal portions.

Transesophageal Echocardiography (TEE) is typically reserved as a problem-solving tool when TTE findings are inconclusive or when more detailed anatomical definition is required prior to an intervention [1]. The proximity of the esophageal probe to the heart provides superior image resolution. Consequently, TEE can often delineate the fistula’s origin, course, and termination site with greater clarity than TTE [1]. In some complex cases, TEE has proven to be the definitive diagnostic modality [21].

Computed Tomography Coronary Angiography (CTCA) is The Modern Gold Standard for Anatomic Delineation of CPAF. Among all the investigations available, CTCA has rapidly grown to be the most reliable imaging technique in the diagnosis of coronary-to-pulmonary artery fistula. Its strengths lie in its non-invasive nature (requiring only intravenous contrast), exceptional spatial and temporal resolution, and powerful post-processing capabilities that allow for multiplanar and 3D volume-rendered reconstructions [26].

CTCA provides unparalleled and detailed information that are essential for therapeutic planning. It can precisely define:

- The coronary artery of origin (e.g., right coronary artery, left anterior descending artery).
- The exact three-dimensional course of the fistulous tract, including its diameter, length, and degree of tortuosity.
- The specific drainage site into the main pulmonary artery or its branches.
- The presence, size, morphology, and location of any associated coronary artery aneurysms or intraluminal thrombus.
- The complex relationship of the fistula to adjacent cardiac chambers, great vessels, and other vital structures.

This comprehensive anatomical information is critical for the multidisciplinary heart team to determine the feasibility and optimal strategy for closure, whether by percutaneous transcatheter techniques or open surgery [34].

It is recommended to set the scan range in such a way to successfully cover the aortic arch beyond the carina level because most drainage sites will be located above the carina (coronary artery—pulmonary artery, coronary artery—other systemic veins or coronary artery—superior vena cava) [2]

CTCA has the advantages of being non-invasive, having high spatial resolution and having the capability of providing images for a 3-dimensional reconstruction of the complex anatomy, which aids in the decision-making process [35].

From an anatomical standpoint, the right coronary artery is the most frequent origin for a coronary artery fistula (50–55% of cases), followed by the left anterior descending artery (LAD) (35–40% of cases) and, lastly, the left circumflex artery (5–20% of cases) [2].

Cardiac Magnetic Resonance (CMR) is the gold standard for functional assessment. While CTCA provides the anatomical map, cardiac magnetic resonance (CMR) is the unparalleled non-invasive tool for defining the functional consequences of the fistula [25]. Its primary role is the precise and reproducible quantification of the shunt. Using a technique called phase-contrast velocity-encoded imaging, CMR can directly measure blood flow volume in the main pulmonary artery (pulmonary flow, or Qp) and the ascending aorta (systemic flow, or Qs) [36]. From these measurements, the pulmonary-to-systemic flow ratio (Qp:Qs) is calculated. A Qp:Qs ratio greater than 1.5:1 is generally considered hemodynamically significant and serves as a key criterion in the decision-making process for intervention [33].

In addition to shunt quantification, CMR using steady-state free precession (SSFP) cine sequences is the accepted gold standard for accurately measuring right and left ventricular end-diastolic and end-systolic volumes, mass, and ejection fraction [25]. This provides a direct and quantitative assessment of the shunt’s impact on cardiac remodeling. Finally, techniques such as late gadolinium enhancement (LGE) can be employed to detect and quantify areas of myocardial fibrosis or scar, which may be the sequelae of chronic ischemia from the coronary steal phenomenon [7].

Below is a summary of the primary diagnostic tools (Table 1).

**Table 1:** Summary of imaging modalities

Modality	Invasiveness	Radiation	Anatomic detail	Functional assessment	Primary role in CPAF Workup
TTE	None	None	Limited; may see proximal vessel dilation and Doppler jet at termination site	Good; assesses chamber size, ventricular function, estimates PA pressure	Initial Screening & Hemodynamic Impact: First-line test to detect shunt and evaluate its consequences
TEE	Minimally Invasive	None	Good; often superior to TTE for visualizing fistula origin and course	Excellent; detailed assessment of valves and adjacent structures	Problem-Solving: Used when TTE is non-diagnostic or for detailed pre-operative assessment
CTCA	Minimally Invasive (IV contrast)	Yes	Excellent (Gold Standard for Anatomy); provides a 3D roadmap of origin, course, termination, and aneurysms	Limited; provides morphology (chamber size) but not direct flow or ischemia data	Definitive Anatomic Diagnosis & Pre-procedural Planning: The go-to test for detailed mapping before intervention
CMR	None	None	Good; can visualize anatomy but with lower spatial resolution than CTCA	Excellent (Gold Standard for Function); quantifies shunt (Qp:Qs), ventricular volumes/mass, and myocardial scar (LGE)	Functional Quantification & Risk Stratification: The best tool to determine if a shunt is hemodynamically significant
CAG	Invasive	Yes	Excellent; high temporal resolution for flow dynamics. Limited by 2D projection	Limited; can be combined with RHC for pressures and FFR for ischemia	Invasive Confirmation & Interventional Guidance: The traditional standard, now primarily used to confirm findings and as the platform for transcatheter closure

TTE, Transthoracic Echocardiography; TEE, Transesophageal Echocardiography; CTCA, Computed Tomography Coronary Angiography; CMR, Cardiac Magnetic Resonance; CAG, Coronary Angiography

### Treatment strategies

Studies regarding the optimal management of CAFs are few, with those referring to CPAF being fewer, including a limited number of patients and having a relatively short follow-up duration. Standardized treatment guidelines for the management of CPAF have not been established but general guideline recommendations regarding the management of CAFs are presented in the 2018 AHA/ACC Guideline for the Management of Adults with Congenital Heart Disease [7] and 2020 ESC Guidelines for the management of adult congenital heart disease [37]

The decision process in managing fistulas (follow-up vs closure) depends on site of origin of the fistula (proximal versus distal) [38], size of the fistula, patient’s symptoms and presence of complication caused by the fistula (premature atherosclerosis, ischemic heart disease, pulmonary hypertension, congestive heart failure, aneurysm, dissection, rupture, thrombosis, endocarditis, etc.), the anatomy of the fistula, the presence of other indications to undergo surgery, the age and preferences of the patient. Balancing the risk to benefit scale in the case of CPAF can be challenging. The main indications for closure of CPAF are clinical symptoms, particularly related to myocardial ischemia, heart failure and pulmonary hypertension, and, in asymptomatic patients the presence of high-flow shunting (to prevent the occurrence of complications) [39].

Patients with large fistulas (the fistula diameter is > 2 times the largest diameter of the coronary vessel not feeding the coronary fistula) irrespective of symptoms and patients with small (the fistula diameter is < 1 times the largest diameter of the coronary vessel not feeding the coronary fistula) to moderate (the fistula diameter is ≥ 1 to 2 times the largest diameter of the coronary vessel not feeding the coronary fistula) fistulas with symptoms or complications related to fistula (ischemia, arrhythmias, congestive heart failure, pulmonary hypertension, aneurysm formation or rupture) should undergo fistula closure (surgical or percutaneous) [7].

A small fistula in an asymptomatic patient usually needs no closure or medical therapy and can be safely managed with regular follow-ups every 3 to 5 years (a watchful waiting approach) [7]. Studies have suggested that small fistulas can slowly increase in size, although most fistulas showed minimal changes over a period of 10 to 15 years [40]. Although fistulas can be benign from a hemodynamic standpoint, they may lead to more severe atherosclerotic disease, focusing the pathophysiologic process at the ostia of the fistula, and this must be considered during follow-ups. Very rarely the fistula can close spontaneously [41].

Intermediate (moderate to large) fistula without symptoms are managed differently depending on the location: in proximal fistula closure is recommended and in distal fistula closure or no-closure with regular follow-ups can be equally used. After closure antiplatelet therapy for at least one year is recommended and, in case of a watchful waiting approach, antiplatelet therapy is recommended indefinitely [42].

The approach of elimination of the fistula (surgical versus transcatheter) depends on the expertise of the physicians involved in the management of the patient and a series of other factors related to the anatomy of the fistula, the presence or absence of associated defects and comorbidities. The occlusion of the fistula must be performed by surgeons or interventionists with training and expertise in congenital heart diseases [7]. Results from the transcatheter and surgical approach show that both have similar rates of success, early morbidity and mortality [43].

Percutaneous intervention requires favorable anatomical characteristics: accessible proximal fistula portion (no ostial lesion or angulation), a narrow distal portion to avoid device embolization into pulmonary artery and no major tortuosity [23] or high perioperative risk profile for open surgery [14]. Surgical closure is preferred for all other fistulas, for large aneurysms or if the patient has other comorbidities that require heart surgery (aortic pathologies, valve replacement, CABG, etc.). Aneurysm formation is the primary indication for open heart surgery because of the risk of rupture. For coronary artery aneurysms, surgery is indicated when the diameter exceeds 30 mm [44].

The most used surgical treatment is fistula ligation [45] and the recurrence rate after CPAF ligation surgery is up to 20–30% [46, 47]. Although surgical closure is associated with low mortality and morbidity, long-term outcome is excellent, and most patients remain asymptomatic [48, 49], closure during cardiac catheterization has become the method of choice and numerous percutaneous catheter techniques have been used (Gianturco coils, interlocking detachable coils, detachable balloons, polyvinyl alcohol foam, double umbrellas, the Amplatzer duct rrhyth, and the Amplatzer vascular plug) [33, 50–54]. The risks associated with transcatheter interventions include myocardial infarction, arrhythmias, coronary artery spasm, fistula dissection and embolization of coils or discs to coronary arteries or other vascular structures [33, 55]. After transcatheter closure, heparin should be started six hours after the procedure and overlapped with an antivitamin K anticoagulant until oral anticoagulation is efficient (international normalized ratio 2–3). Patients should receive an oral anticoagulant a period of 6 to 12 months [38] and after this period, they should receive antiplatelets indefinitely.

Regarding the optimal medical treatment used in CPAF there is not enough data to make specific recommendations. Drugs such as beta-blockers, calcium channel blockers, anti-hyperlipidemic agents, and antiplatelet agents were used without guideline indications [14]. It is recommended to treat the complications that arise (myocardial ischemia, heart failure, pulmonary hypertension, rrhythmias, endocarditis) according to current practice guidelines [7, 33, 50–54]. Although the risk of endocarditis slightly higher, endocarditis prophylaxis is not routinely indicated in this condition [56].

In conclusion the clinical course of small CPAF is usually benign and the recommended approach is periodical clinical and imaging evaluation as they may slowly increase in size with advancing age and changes in systemic blood pressure and aortic compliance. Medium or large fistula are associated with long-term complications (myocardial ischemia, arrhythmia, heart failure, endocarditis, etc.). If the size of the fistula and the shunt is associated with symptoms or complications then occlusion of the fistula, optimal medical treatment of complications and comorbidities and regular long-term follow-up is recommended [7, 37].

To contextualize our findings and provide a broader overview of current therapeutic approaches, we have summarized a selection of recently published case reports on coronary-to-pulmonary artery fistulas in Table 2.

**Table 2:** Summary of recent case reports on coronary-to-pulmonary artery fistulas and management strategies

References	Year	Journal	Type of Fistula	Management
Namura et al. [57]	2024	*Eur Heart J Case Rep*	Giant RCA + LAD → PA fistulas	Coil embolization (two-stage)
Takahashi et al. [58]	2024	*Eur Heart J Case Rep*	Giant aneurysmal LAD → PA fistula (thrombosed spontaneously)	Coil embolization
Sato Namura et al. [57]	2024	*Eur Heart J Case Rep*	Giant CPFs → PA (significant left–right shunt)	Coil embolization
Liu et al. [59]	2024	*J Cardiothorac Surg*	Multiple CPAFs with coronary aneurysm + Vieussens’ ring	Surgical ligation and aneurysm resection
Vivekanandan et al. [60]	2024	*J Surg Case Rep*	Coronary–PA fistula + critical LAD stenosis	Surgical ligation + CABG
Rubimbura et al. [61]	2021	*Front Cardiovasc Med*	Bilateral coronary → PA fistulas	Coils + vascular plug after failed plug
Moriyama et al. [62]	2024	*Eur Heart J Case Rep*	Vieussens’ arterial ring + aneurysm + RCA–PA fistula	Surgical closure
Zhou Ting et al. [63]	2022	*Frontiers in Cardiovascular Medicine*	Multiple LAD–PA fistulas with ASD and bicuspid pulmonary valve	Surgical repair during open-heart surgery
Shibata et al. [64]	2022	*J Am Coll Cardiol Case Rep*	Coronary–PA fistula presenting with tamponade due to aneurysm	Surgical repair
Quatrini et al. [65]	2007	Cardiovasc Ultrasound	LAD → PA fistula in adolescent	Conservative evaluation
Figueroa-Casanova et al. [66]	2022	*Cirugia Cardiovasc*	75%stenosis of LAD + PA fistula with large aneurysm	Surgical repair + CABG
Dadkhah-Tirani et al. [43]	2013	Am J Case Rep	LAD to the main pulmonary artery fistula + RCA blockage and significant stenoses on the LAD and LCX	Surgical repair + CABG
Kamal et al. [67]	2022	Int J Surg Case Rep	Proximal LAD to main PA fistula and severe stenosis in LAD	Surgical repair + CABG

As demonstrated in the cases summarized in Table 2, the management of coronary-to-pulmonary artery fistulas (CPAFs) is highly individualized and primarily dictated by the fistula’s anatomy and the presence of associated cardiac conditions. A notable trend is the use of surgical intervention—typically ligation with or without coronary artery bypass grafting (CABG)—in complex cases involving significant coronary artery stenosis or large aneurysms. In contrast, percutaneous coil embolization is a frequently employed strategy for less complex fistulas, sometimes performed in a staged manner. Conservative management may be considered in select asymptomatic cases. The heterogeneity of these approaches underscores the need for a tailored strategy based on comprehensive diagnostic imaging.

**Fig. 1:**
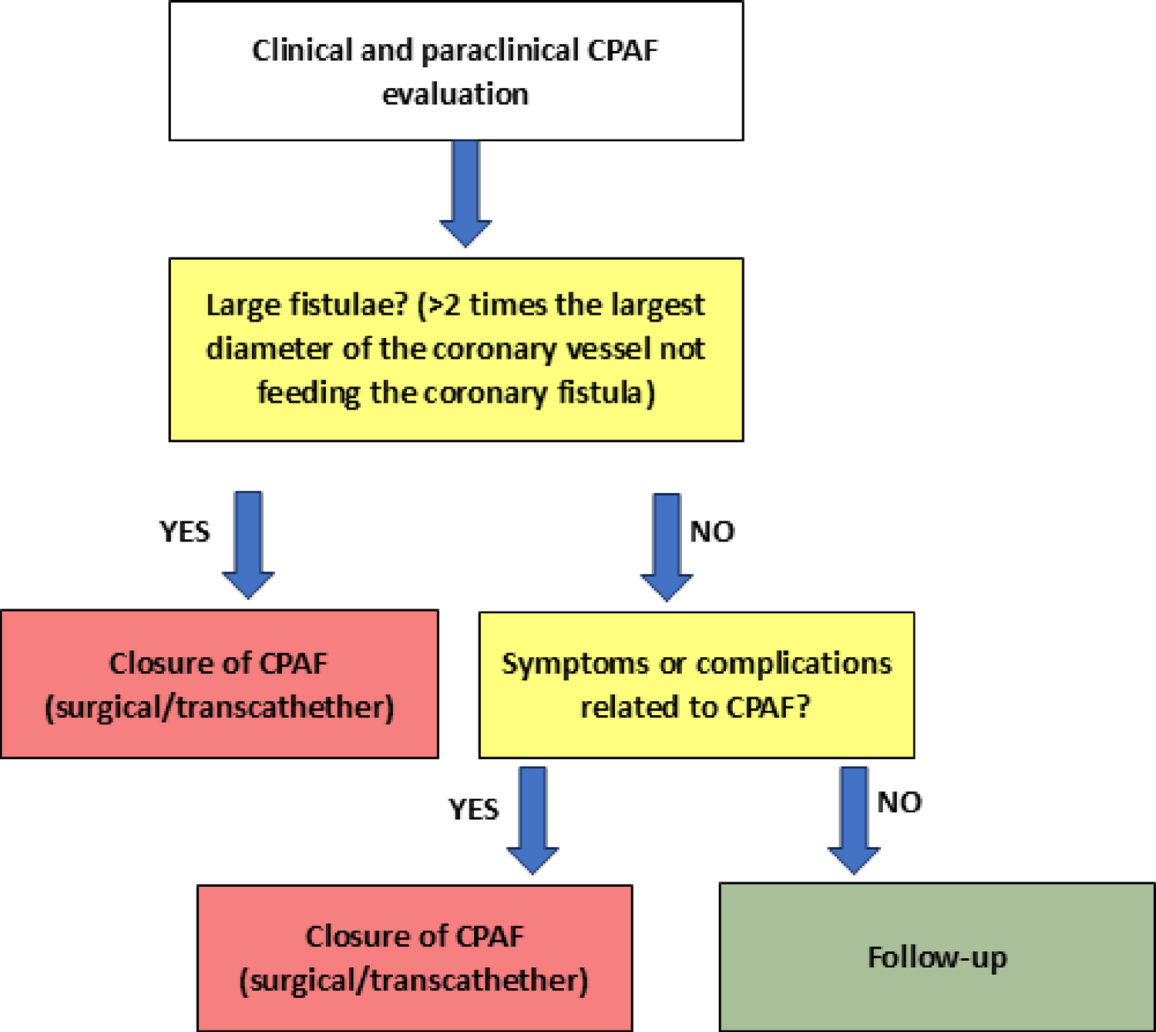
Stepwise management algorithm for coronary-to-pulmonary artery fistula (CPAF)

To synthesize the current management strategies discussed in the literature, we propose a practical, stepwise algorithm. This decision-making flowchart, presented in Fig. 1, stratifies patients based on key anatomical features, such as fistula size, and the presence of clinical symptoms or complications to guide the choice between intervention and conservative follow-up.

## Case presentation

A 48-year-old male patient presented to the Emergency Department (ED) with anterior thoracic pain which radiated to the interscapular region. The pain wasn’t linked to exertion; it was recurrent and each episode lasted approximately 30 to 40 min.

From the patient’s medical history, we noted that he was known with gastric ulcer and cervical spondylosis. Also, he had two negative ECG exercise tests in the two years prior to admission. The patient was an ex-smoker.

### Physical examination

On admission, blood pressure was 130/80 mmHg bilaterally, heart rate was 65, rhythmic. On cardiac auscultation no pathological sounds or murmurs were found. Pulmonary auscultation was normal.

### Diagnostic assessment

**Fig. 2:**
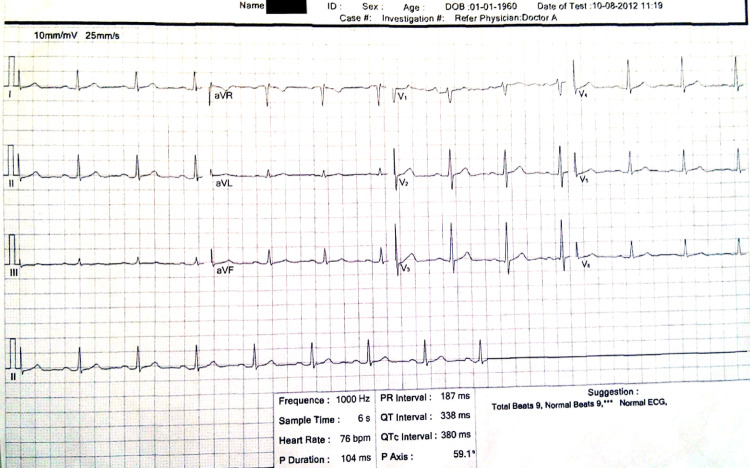
Patient ECG performed in the ED

An ECG was performed, which showed sinus rhythm, QRS axis at 50 degrees and no ST-T segment abnormalities (Fig. 2).

**Fig. 3:**
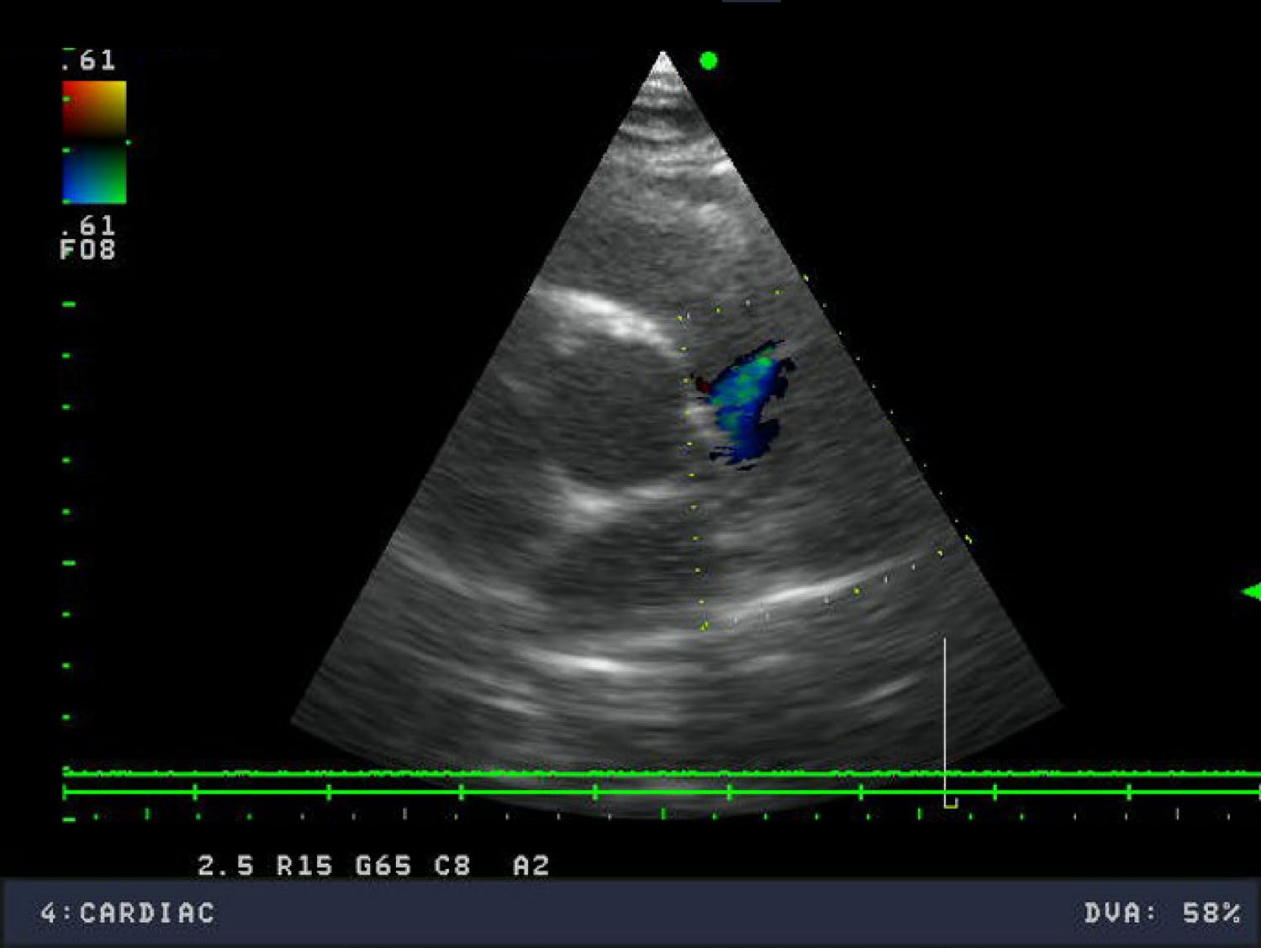
Echocardiography (parasternal short axis view at the level of the great vessels) showing abnormal flow into the pulmonary artery

Echocardiography was performed next and it revealed the presence of an abnormal, turbulent flow in the pulmonary artery that seemed to originate from the aorta or the coronary artery (Fig. 3). Otherwise, the exam was unremarkable: preserved ejection fraction, right and left heart dimensions within normal limits, and the pulmonary-to-systemic flow ratio (Qp:Qs) was 1:1.

**Fig. 4:**
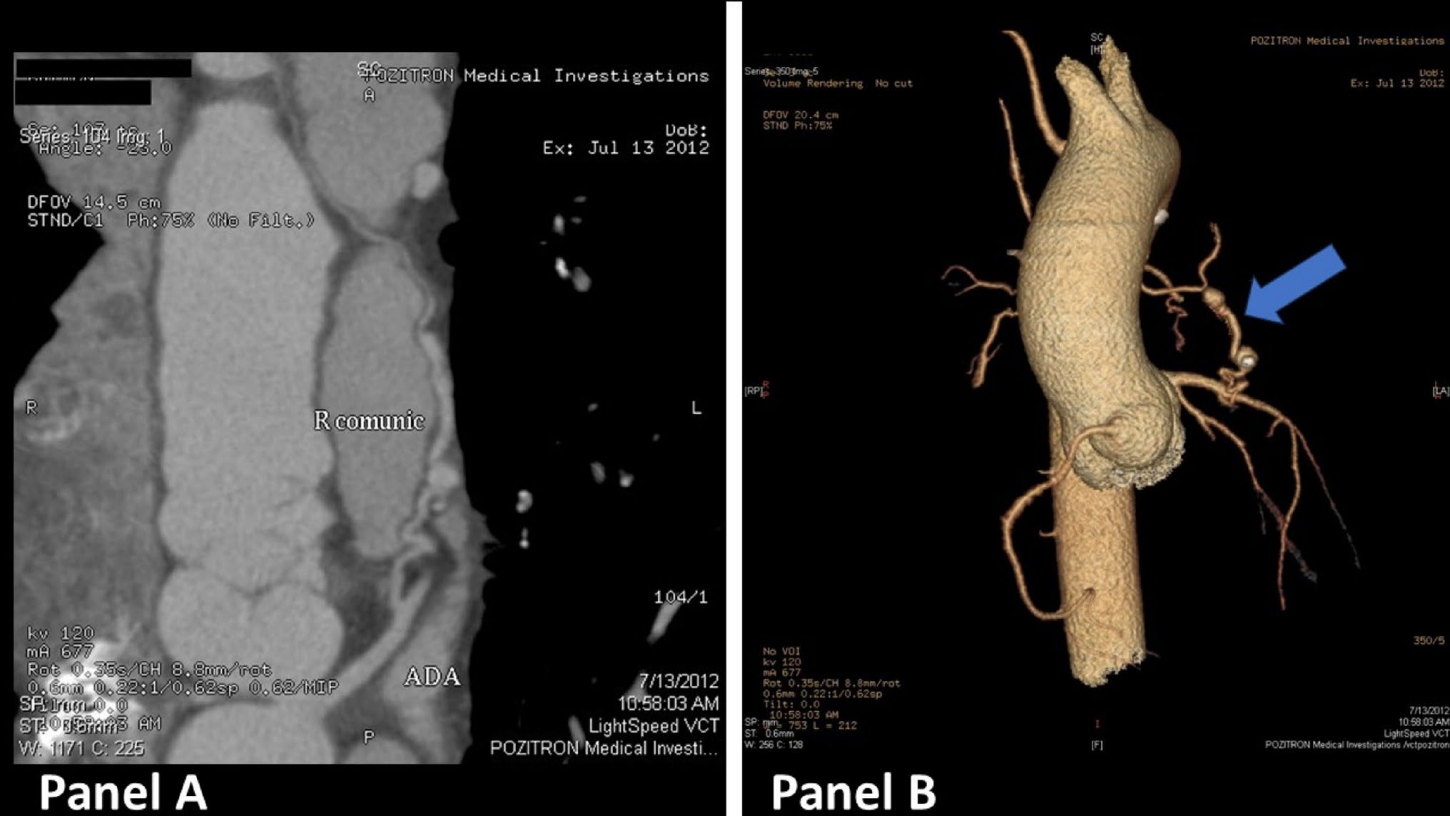
**A** The fistula that connects the LAD with the pulmonary artery trunk. **B** 3D reconstruction showing the fistula (blue arrow)

**Fig. 5:**
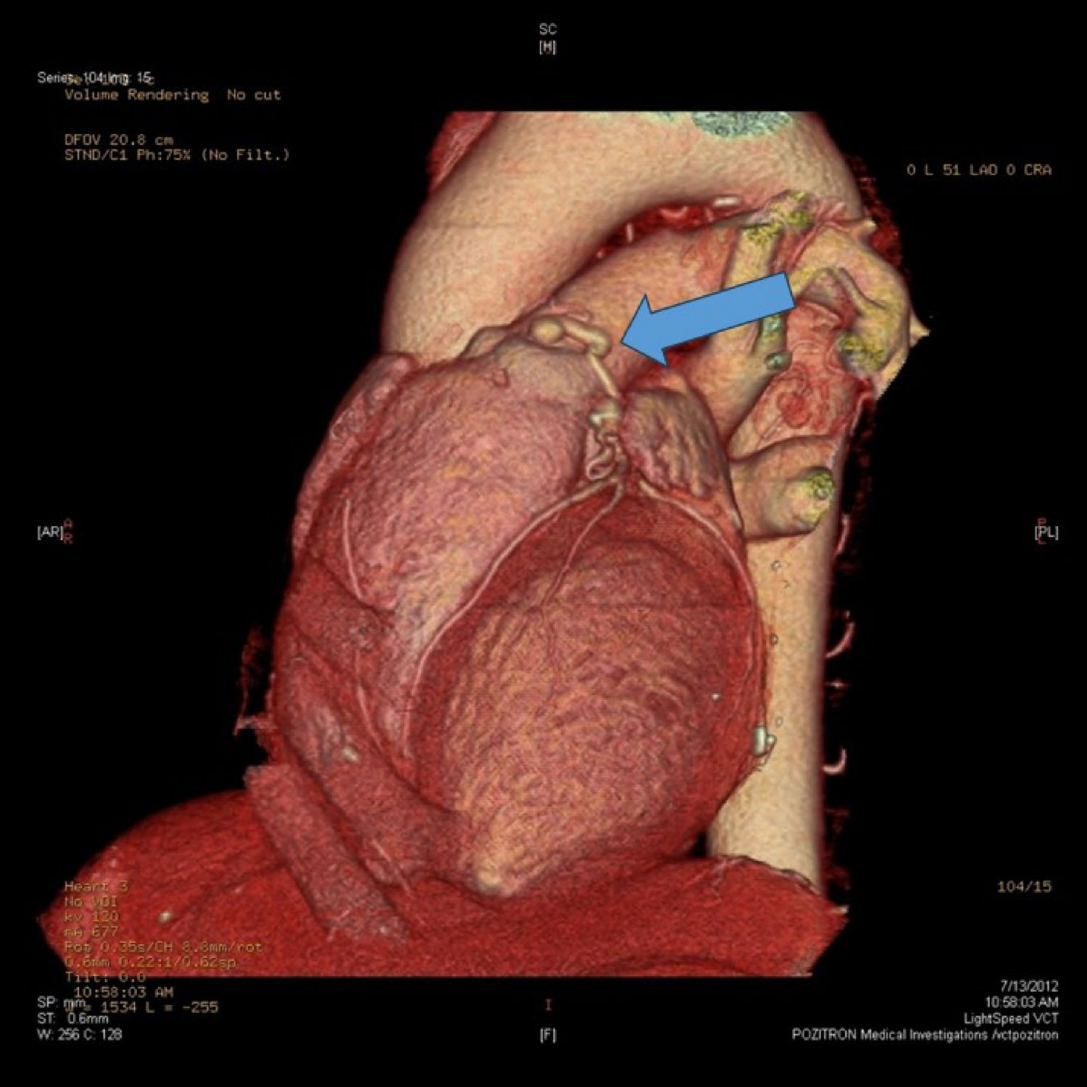
3D reconstruction showing the CPAF (blue arrow) and its anatomical relations

The patient was admitted and further investigations were performed. CT coronary angiography revealed an abnormal left anterior descending artery with a fistulous drainage to the main pulmonary artery (Figs. 4 and 5).

**Fig. 6:**
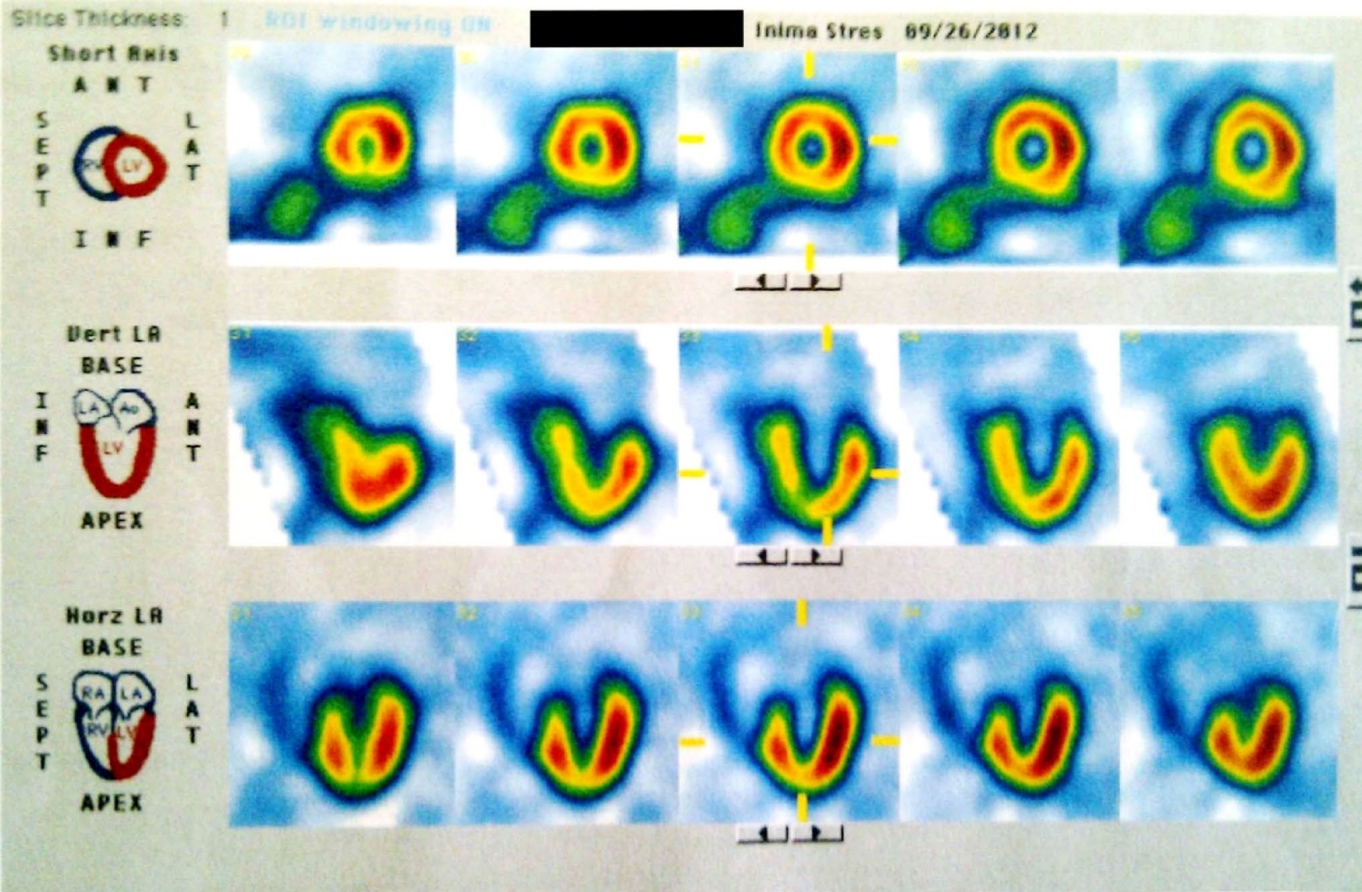
Myocardial perfusion scan demonstrating no perfusion defects in the anterior wall

The patient had two prior ECG exercise tests which revealed no issues. As such, we decided to investigate the possibility of myocardial ischemia through myocardial perfusion scintigraphy. The scan revealed hypoperfusion in the inferior segments of the left ventricular wall, which are typically supplied by the right coronary artery. No myocardial perfusion defects were observed in the territory served by the left anterior descending artery (Fig. 6).

**Fig. 7:**
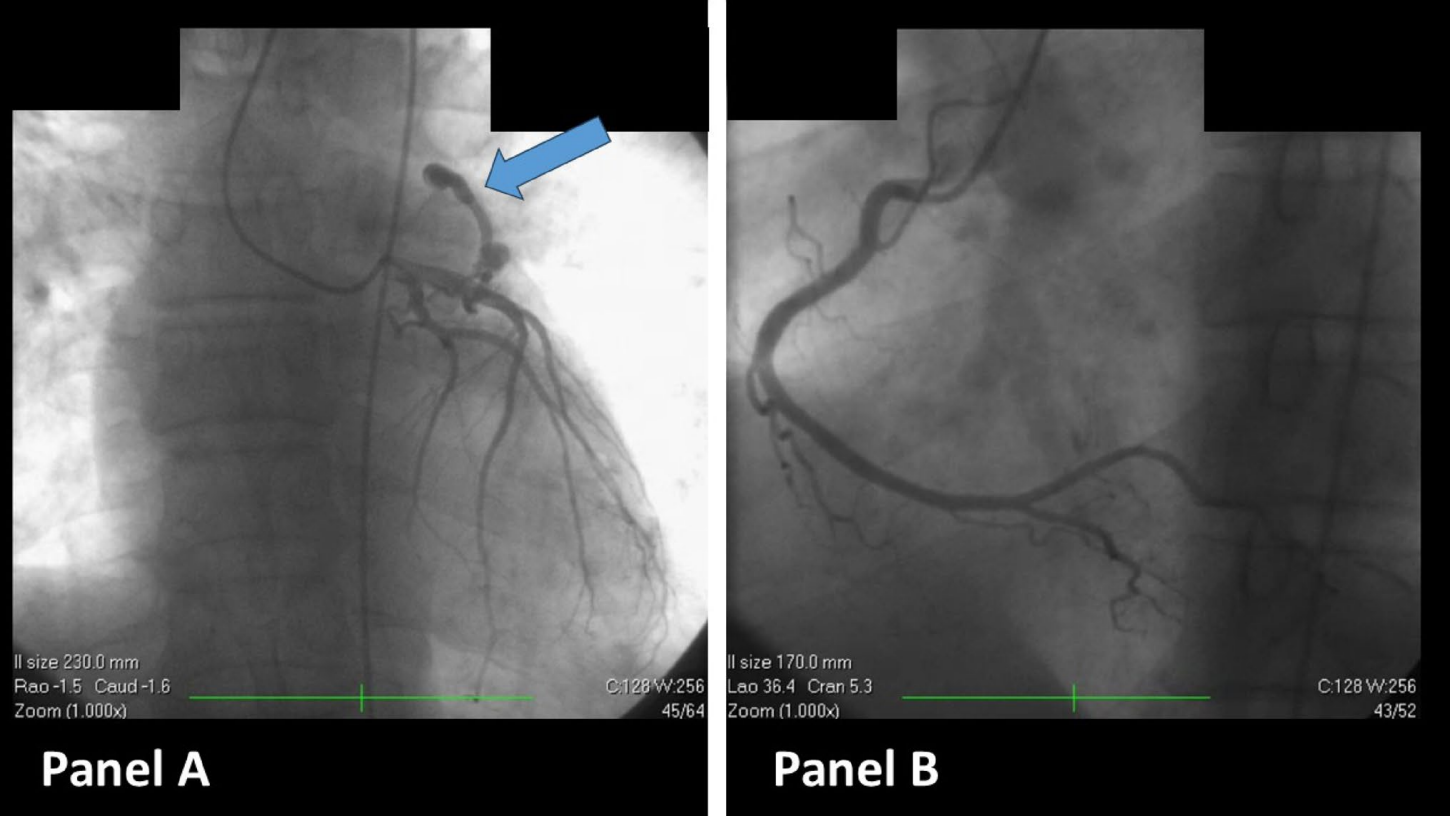
Coronarography. **A** CPAF arising from proximal LAD; otherwise, normal LAD; **B** RCA with no significant atherosclerotic lesions

The coronarography showed the abnormal flow through the LAD-pulmonary artery fistula, but otherwise was normal, with no significant atherosclerotic lesions (Fig. 7).

**Fig. 8:**
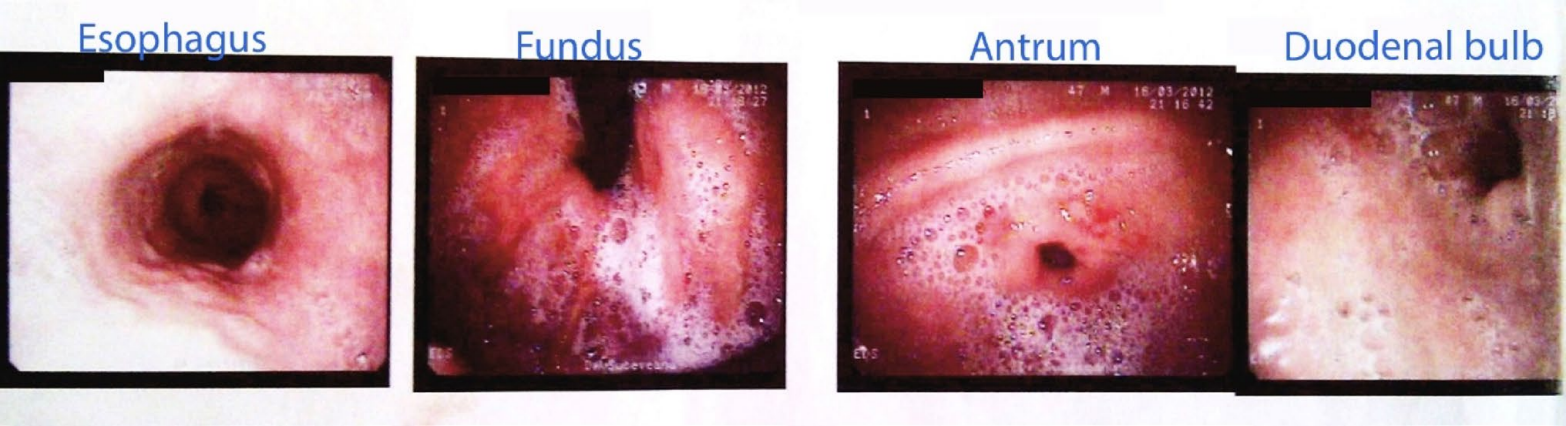
Upper endoscopy revealed small ulcers in the pyloric region

After we successfully excluded a cardiac origin for the pain experienced by the patient, we focused on other possible causes. Given the patient’s history of gastric ulcer, a gastroenterological consult was solicitated and an upper endoscopy was performed. The investigation revealed small ulcers in the pyloric region (Fig. 8).

### Interventions

The patient received peptic ulcer treatment and his symptoms subsided.

### Follow-up

The patient was followed-up in an ambulatory setting, at 3, 6 and 12 months from the index event. The patient had been symptom free in between visits. Echocardiography was performed at each visit (including measuring Qp:Qs), with no new lesions and with a constant Qp:Qs ratio of 1:1. An ECG exercise test was performed at the 6 month mark, with normal results.

We decided to abstain from medical intervention in the case of our patient because he was asymptomatic, with a normal ratio of pulmonary to systemic flow, no pulmonary artery enlargement, normal pulmonary artery pressure and with no evidence of myocardial ischemia in the territory served by the left anterior descending artery. The patient was stable, asymptomatic at each follow up. The inferior hypoperfusion was considered an incidental finding, given that the patient’s symptoms were gastrointestinal in origin and resolved completely with ulcer treatment.

## Conclusion and take-home messages

CPAF are rare anomalies that, while often asymptomatic, can lead to severe complications depending on their size and hemodynamic impact. The case presented here illustrates the importance of thorough diagnostic evaluation in patients with CPAF to assess potential symptoms or complications and alternative diagnoses. Although the patient experienced recurrent thoracic pain, thorough investigations revealed the CPAF had a minimal hemodynamic impact and was not the underlying cause for the patient’s symptoms. Regarding the CPAF, conservative management was chosen, supporting the principle that watchful waiting could be a safe and appropriate approach for small, asymptomatic fistulas without significant hemodynamic compromise.

### Take-home messages

1. CPAFs are rare but should be considered in patients presenting with unexplained chest pain or right heart failure secondary to pulmonary hypertension, in the absence of other causes for the patient’s symptoms.
2. Diagnostic imaging is integral: CT coronary angiography provides a non-invasive, high-resolution, 3D view that is particularly useful for visualizing the complex anatomy of CPAFs and aiding in treatment planning.
3. Conservative management is viable for small, asymptomatic CPAFs with no significant shunt flow or myocardial ischemia. Regular follow-up represents a key component of management to monitor for potential changes.
4. Surgical or percutaneous intervention is generally recommended for CPAFs with larger shunts, significant coronary steal syndrome, aneurysmal degeneration, or associated pulmonary hypertension.
5. An individualized approach is frequently appropriate: decisions should weigh the patient’s symptoms, hemodynamic impact, and anatomical suitability for percutaneous versus surgical intervention.

### Limitations

This is a single case report with a follow-up of one year, which limits the generalizability of our observations. While serial imaging showed stability, longer-term follow-up would be required to confirm the absence of progression. Furthermore, our conclusions regarding the safety of conservative management apply only to similar cases without significant hemodynamic compromise.

## Data Availability

No datasets were generated or analysed during the current study.
